# Development and validation of a nomogram to predict overall survival in patients with glioma: a population-based study

**DOI:** 10.18632/aging.205967

**Published:** 2024-07-05

**Authors:** Wei Huang, Yuhe Lei, Xiongbin Cao, Gengrui Xu, Xiaokang Wang

**Affiliations:** 1 Department of Internal Medicine, Shenzhen Longhua District Maternity and Child Healthcare Hospital, Shenzhen 518109, China; 2 Department of Pharmacy, Shenzhen Hospital of Guangzhou University of Chinese Medicine, Shenzhen 518034, China; 3 Department of Neurology, Shenzhen Longhua District Central Hospital, Shenzhen 518110, China; 4 Department of Pharmacy, Shenzhen Longhua District Central Hospital, Shenzhen 518110, China

**Keywords:** glioma, nomogram, prognostic model, overall survival, SEER

## Abstract

Aim: The objective is to investigate the prognostic factors associated with gliomas and to develop and assess a predictive nomogram model connected to survival that may serve as an additional resource for the clinical management of glioma patients.

Method: From 2010 to 2015, participants included in the study were chosen from the Surveillance Epidemiology and End Results (SEER) database. Gliomas were definitively diagnosed in each of them. They were divided into the training group and the validation cohort at random (7/3 ratio) using a random number table. To identify the independent predictive markers for overall survival (OS), Cox regression analysis was utilized. Subsequently, the training cohort’s survival-related nomogram predictive model for OS was created by incorporating the fundamental patient attributes. Following that, the training cohort’s model underwent internal validation. The nomogram model’s authenticity and reliability were assessed through the computation of receiver operating characteristic (ROC) curves and concordance index (C-index). To evaluate the degree of agreement between the observed and predicted values in the training and validation cohorts, calibration plots were created.

Result: Age, primary site, histological type, surgery, chemotherapy, marital status, and grade were the independent predictive factors for OS in the training cohort, according to Cox regression analysis. Moreover, the nomogram model for predicting 1-year, 3-year, and 5-year OS was built using these variables. The C-indexes of OS for glioma patients in the training cohort and internal validation cohort were found to be 0.779 (95% CI=0.769-0.789) and 0.776 (95% CI=0.760-0.792), respectively, according to the results. The ROC curves also demonstrated good discrimination. Additionally, calibration plots demonstrated a fair amount of agreement.

Conclusions: In summary, the nomogram prediction model of OS demonstrated a moderate level of reliability in its predictive performance, offering valuable reference data to enable doctors to quickly and easily determine the survival likelihood of patients with gliomas.

## INTRODUCTION

About one-third of intracranial tumors are gliomas, the most frequent malignant tumor inside the brain that arises from the central nervous system (CNS) [1, 2]. According to molecular staging, tumor cell anisotropy, mesenchymal degeneration, nuclear fission activity, degree of microvascular proliferation, and degree of tumor necrosis, the World Health Organization divides gliomas into low-grade and high-grade gliomas [3, 4]. Currently, the main treatment options for patients with glioma include surgery, radiation therapy, chemotherapy, and follow-up observation [5–7]. Of these, surgery is currently recognized as the first line of treatment [8, 9]. Few studies have been reported on the clinical characteristics and prognostic factors related to glioma patients, and in addition, few studies have been published on the prediction models associated with the prognosis of gliomas patients. In order to create a useful prognostic prediction model for glioma patients, we therefore created a nomogram for predicting overall survival (OS).

We collected the data from the Surveillance, Epidemiology, and End Results (seer) Database since the development of such a predictive model necessitates a sizable clinical database with a big sample size, multicenter, and high confidence. Publicly available oncology data from 28% of the US population are bundled in the Seer database, which also includes information on patient demographics, primary tumor sites, treatment details, and survival rates [10]. The nomogram is a visual graphical presentation of complex mathematical formulas [11, 12]. By integrating different clinical variables, nomograms can create statistical predictive models that can be used to help determine the risk of cancer recurrence or death [13]. This work aims to provide an accurate estimate of OS and risk assessment for glioma patients undergoing additional treatment by creating a complete and useful nomogram for glioma patients utilizing the SEER database.

## RESULTS

### Features of the patients

**Figure 1:**
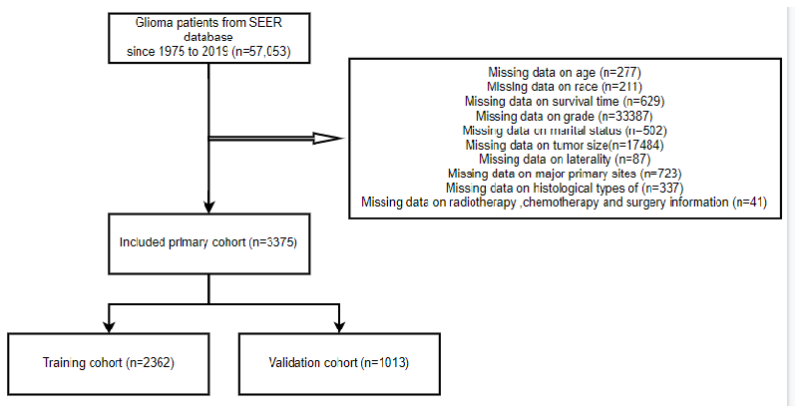
The flow diagram of how cases were selected from the SEER database.

3,375 glioma patients in all, who met the selection criteria, were gathered for this investigation from the SEER database. R program randomly allocated 2,362 patients (69.99%) to the training set and 1,013 patients (30.01%) to the test set, divided the patients into two groups based on the proportion of 7:3 (Figure 1). Among the training cohort, 2,130 patients (90.2%) were White, 1,371 patients (58.0%) were male, and 1,548 patients (65.5%) were between the ages of 40 and 60. Regarding the validation cohort, 581 (57.4%) patients were male, and 639 (63.1%) patients were aged 40-60 years old. Table 1 displays the particular baseline clinicopathological characteristics.

**Table 1:** Summary of clinicopathologic features and treatments of patients with glioma.

	**All**	**Training**	**Test**	**p-value**
	**N=3375**	**N=2362**	**N=1013**	
Age				0.591
<18	30 (0.89%)	21 (0.89%)	9 (0.89%)	
18-65	2187 (64.8%)	1548 (65.5%)	639 (63.1%)	
66-79	892 (26.4%)	611 (25.9%)	281 (27.7%)	
≥80	266 (7.88%)	182 (7.71%)	84 (8.29%)	
Race				0.677
White	3047 (90.3%)	2130 (90.2%)	917 (90.5%)	
Black	138 (4.09%)	101 (4.28%)	37 (3.65%)	
Other	190 (5.63%)	131 (5.55%)	59 (5.82%)	
Gender				0.739
Male	1952 (57.8%)	1371 (58.0%)	581 (57.4%)	
Female	1423 (42.2%)	991 (42.0%)	432 (42.6%)	
Laterality				0.529
Left	1472 (43.6%)	1025 (43.4%)	447 (44.1%)	
Right	1518 (45.0%)	1075 (45.5%)	443 (43.7%)	
Not a paired site	385 (11.4%)	262 (11.1%)	123 (12.1%)	
Site				0.226
Frontal lobe	1334 (39.5%)	951 (40.3%)	383 (37.8%)	
Temporal lobe	830 (24.6%)	577 (24.4%)	253 (25.0%)	
Parietal lobe	602 (17.8%)	409 (17.3%)	193 (19.1%)	
Occipital lobe	134 (3.97%)	102 (4.32%)	32 (3.16%)	
Overlapping lesion	475 (14.1%)	323 (13.7%)	152 (15.0%)	
Histological				0.053
Mixed glioma	158 (4.68%)	107 (4.53%)	51 (5.03%)	
Astrocytoma	795 (23.6%)	569 (24.1%)	226 (22.3%)	
Glioblastoma	2021 (59.9%)	1386 (58.7%)	635 (62.7%)	
Oligodendroglioma	401 (11.9%)	300 (12.7%)	101 (9.97%)	
Surgery				0.898
Yes	2768 (82.0%)	1939 (82.1%)	829 (81.8%)	
No	607 (18.0%)	423 (17.9%)	184 (18.2%)	
Radiotherapy				0.819
Yes	2642 (78.3%)	1846 (78.2%)	796 (78.6%)	
No	733 (21.7%)	516 (21.8%)	217 (21.4%)	
Radiation sequence with surgery:				0.924
Prior to surgery	15 (0.44%)	10 (0.42%)	5 (0.49%)	
After surgery	2204 (65.3%)	1542 (65.3%)	662 (65.4%)	
Before and after	8 (0.24%)	5 (0.21%)	3 (0.30%)	
Unknown	1148 (34.0%)	805 (34.1%)	343 (33.9%)	
Chemotherapy				0.85
Yes	2305 (68.3%)	1616 (68.4%)	689 (68.0%)	
No	1070 (31.7%)	746 (31.6%)	324 (32.0%)	
Status				0.047
Alive	644 (19.1%)	472 (20.0%)	172 (17.0%)	
Dead	2731 (80.9%)	1890 (80.0%	841 (83.0%)	
Marital				0.982
Married	2199 (65.2%)	1534 (64.9%)	665 (65.6%)	
Single	571 (16.9%)	403 (17.1%)	168 (16.6%)	
Separated/Divorced	315 (9.33%)	221 (9.36%)	94 (9.28%)	
Widowed	290 (8.59%)	204 (8.64%)	86 (8.49%)	
Size (mm)				0.326
<26	780 (23.1%)	542 (22.9%)	238 (23.5%)	
27-44	1336 (39.6%)	920 (39.0%)	416 (41.1%)	
>44	1259 (37.3%)	900 (38.1%)	359 (35.4%)	
Grade				0.688
Grade I+II	278 (8.24%)	198 (8.38%)	80 (7.90%)	
Grade III+IV	3097 (91.8%)	2164 (91.6%)	933 (92.1%)	

### Survival prognostic factor analysis using clinical factors

**Figure 2:**
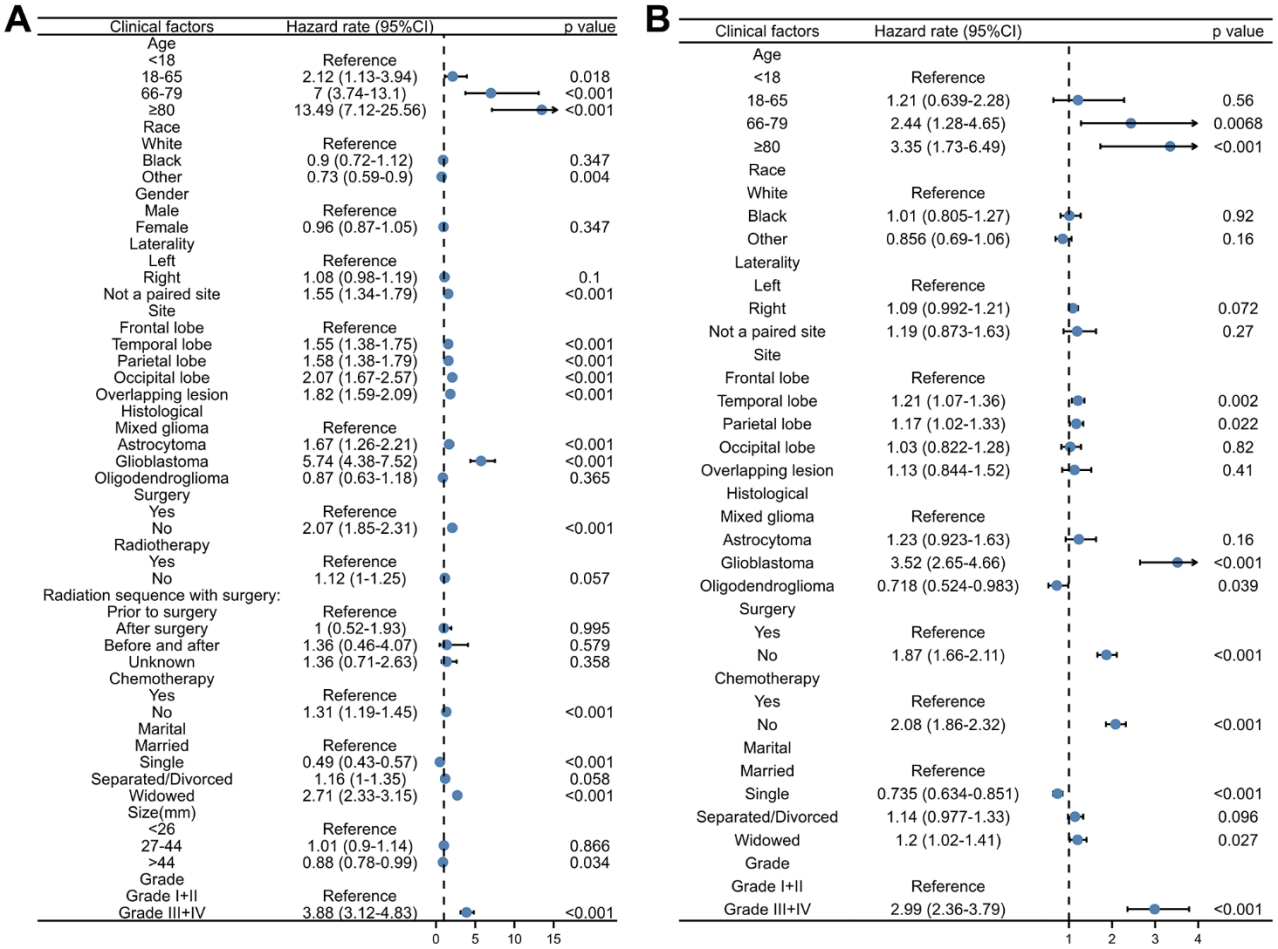
**The forest plot of the univariate and multivariate Cox regression analysis of OS in the training cohort.** (**A**) Univariate Cox regression analysis. (**B**) Multivariate Cox regression analysis.

The univariate analysis results for the training cohort showed that the following factors were related to glioma patient survival: age, race, laterality, primary site, histological type, surgery, chemotherapy, marital status, and grade (all P<0.05). But there was no correlation between tumor size, treatment, or sex and survival (all P>0.05). Age, primary site, histological type, surgery, chemotherapy, marital status, grade, and other factors (all P<0.05) were linked in the multivariate analysis to lower overall survival (OS) rates for glioma patients. Tables 2, 3 display the findings of the univariate and multivariate analyses. To further visualize the findings, forest plots were created (Figure 2).

**Table 2:** Univariate Cox regression analysis of overall survival in the training cohort.

	**Hazard rate (95%CI)**	**p-value**	
Age			
<18	1.00		
18-65	2.12 (1.13-3.94)		**0.018**
66-79	7 (3.74-13.1)	**<0.001**	
≥80	13.49 (7.12-25.56)	**<0.001**	
Race			
White	1.00		
Black	0.9 (0.72-1.12)		0.347
Other	0.73 (0.59-0.9)		**0.004**
Gender			
Male	1.00		
Female	0.96 (0.87-1.05)		0.347
Laterality			
Left	1.00		
Right	1.08 (0.98-1.19)		0.1
Not a paired site	1.55 (1.34-1.79)	**<0.001**	
Site			
Frontal lobe	1.00		
Temporal lobe	1.55 (1.38-1.75)	**<0.001**	
Parietal lobe	1.58 (1.38-1.79)	**<0.001**	
Occipital lobe	2.07 (1.67-2.57)	**<0.001**	
Overlapping lesion	1.82 (1.59-2.09)	**<0.001**	
Histological			
Mixed glioma	1.00		
Astrocytoma	1.67 (1.26-2.21)	**<0.001**	
Glioblastoma	5.74 (4.38-7.52)	**<0.001**	
Oligodendroglioma	0.87 (0.63-1.18)		0.365
Surgery			
Yes	1.00		
No	2.07 (1.85-2.31)	**<0.001**	
Radiotherapy			
Yes	1.00		
No	1.12 (1-1.25)		0.057
Radiation sequence with surgery:			
Prior to surgery	1.00		
After surgery	1 (0.52-1.93)		0.995
Before and after	1.36 (0.46-4.07)		0.579
Unknown	1.36 (0.71-2.63)		0.358
Chemotherapy			
Yes	1.00		
No	1.31 (1.19-1.45)	**<0.001**	
Marital			
Married	1.00		
Single	0.49 (0.43-0.57)	**<0.001**	
Separated/Divorced	1.16 (1-1.35)		0.058
Widowed	2.71 (2.33-3.15)	**<0.001**	
Size (mm)			
<26	1.00		
27-44	1.01 (0.9-1.14)		0.866
>44	0.88 (0.68-1.09)		0.054
Grade			
Grade I+II	1.00		
Grade III+IV	3.88 (3.12-4.83)	**<0.001**	

**Table 3:** Multivariate Cox regression analysis of overall survival in the training cohort.

	**Hazard rate (95%CI)**	**p-value**	
Age			
<18	1.00		
18-65	1.21 (0.639-2.28)		0.56
66-79	2.44 (1.28-4.65)		**0.0068**
≥80	3.35 (1.73-6.49)	**<0.001**	
Race			
White	1.00		
Black	1.01 (0.805-1.27)		0.92
Other	0.856 (0.69-1.06)		0.16
Laterality			
Left	1.00		
Right	1.09 (0.992-1.21)		0.072
Not a paired site	1.19 (0.873-1.63)		0.27
Site			
Frontal lobe	1.00		
Temporal lobe	1.21 (1.07-1.36)		**0.002**
Parietal lobe	1.17 (1.02-1.33)		**0.022**
Occipital lobe	1.03 (0.822-1.28)		0.82
Overlapping lesion	1.13 (0.844-1.52)		0.41
Histological			
Mixed glioma	1.00		
Astrocytoma	1.23 (0.923-1.63)		0.16
Glioblastoma	3.52 (2.65-4.66)	**<0.001**	
Oligodendroglioma	0.718 (0.524-0.983)		**0.039**
Surgery			
Yes	1.00		
No	1.87 (1.66-2.11)	**<0.001**	
Chemotherapy			
Yes	1.00		
No	2.08 (1.86-2.32)	**<0.001**	
Marital			
Married	1.00		
Single	0.735 (0.634-0.851)	**<0.001**	
Separated/Divorced	1.14 (0.977-1.33)		0.096
Widowed	1.2 (1.02-1.41)		**0.027**
Grade			
Grade I+II	1.00		
Grade III+IV	2.99 (2.36-3.79)	**<0.001**	

### Construction and validation of the nomogram

**Figure 3:**
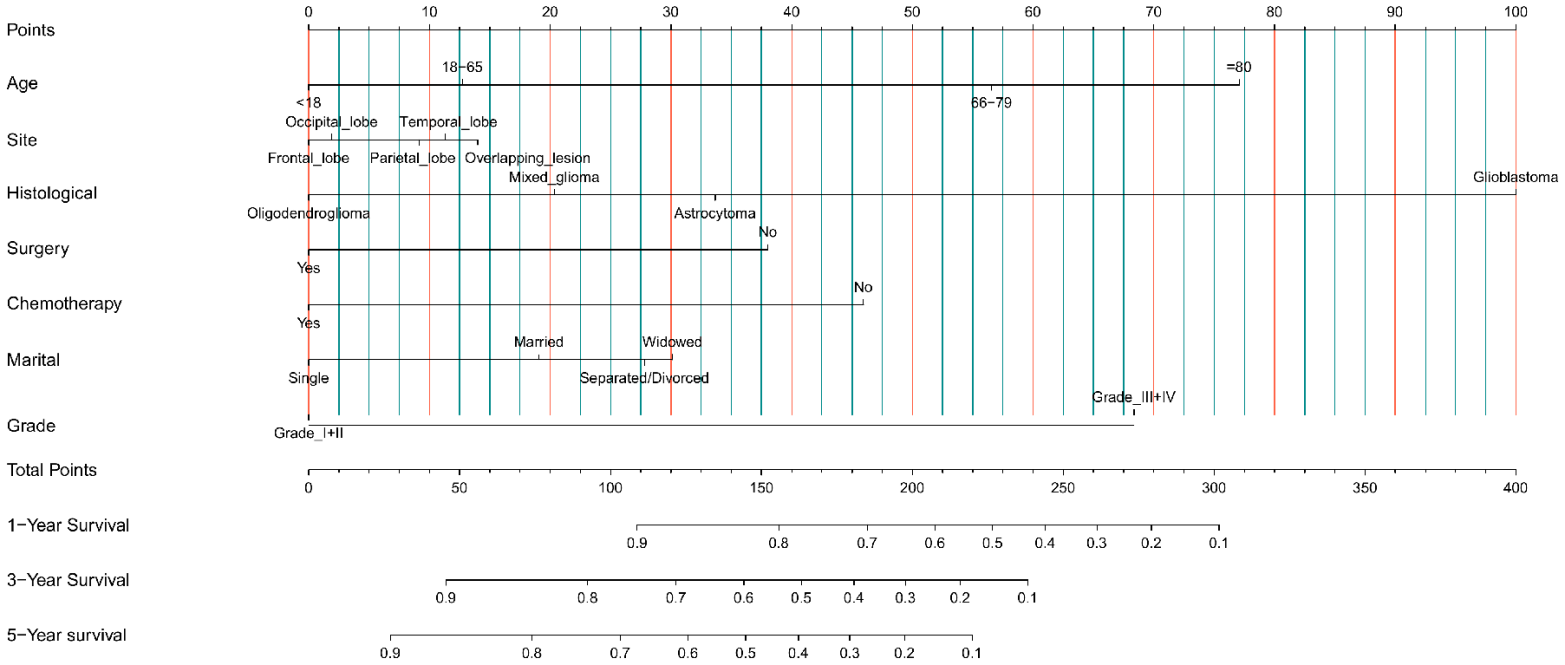
Nomogram prediction model of 1-year, 3-year, and 5-year OS rates in the training cohort.

We created a nomogram based on each of the aforementioned independent OS-related factors from the univariate and multivariate Cox regression analysis in order to forecast the OS of glioma patients. Age, primary site, histological type, surgery, chemotherapy, marital status, and grade are among the independent factors. Every independent prognostic factor was given a final score via the nomogram. A weighted total score derived from each variable was used to determine the 1-, 3-, and 5-year OS prognosis of glioma patients. As illustrated in Figure 3, protective variables for glioma patients include younger age, frontal lobe primary site, histological type of oligodendroglioma, receiving surgery, receiving chemotherapy, and marital status of single and low-grade gliomas. Older age, the primary site of an overlapping lesion, the histological type of glioblastoma, not undergoing chemotherapy or surgery, being separated from their spouse, and high-grade glioma were all associated with a poor prognosis for glioma patients.

**Figure 4:**
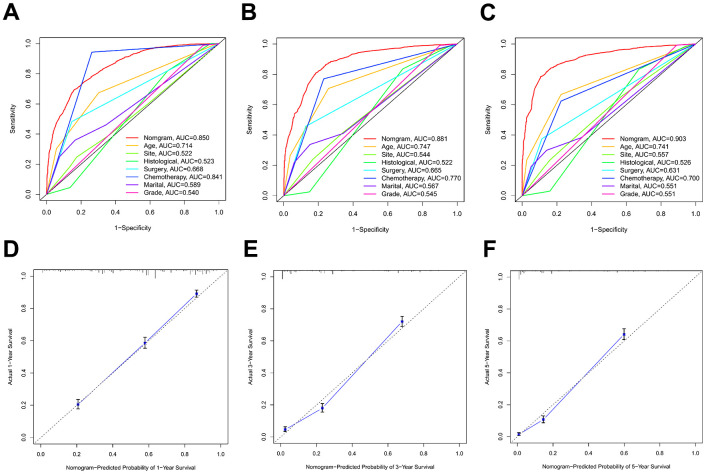
**The ROC curves and calibration plots in the training cohort.** (**A**–**C**) ROC curves of 1-year, 3-year, and 5-year OS rates in the training cohort. (**D**–**F**) Calibration plots of 1-year, 3-year, and 5-year OS rates in the training cohort.

**Figure 5:**
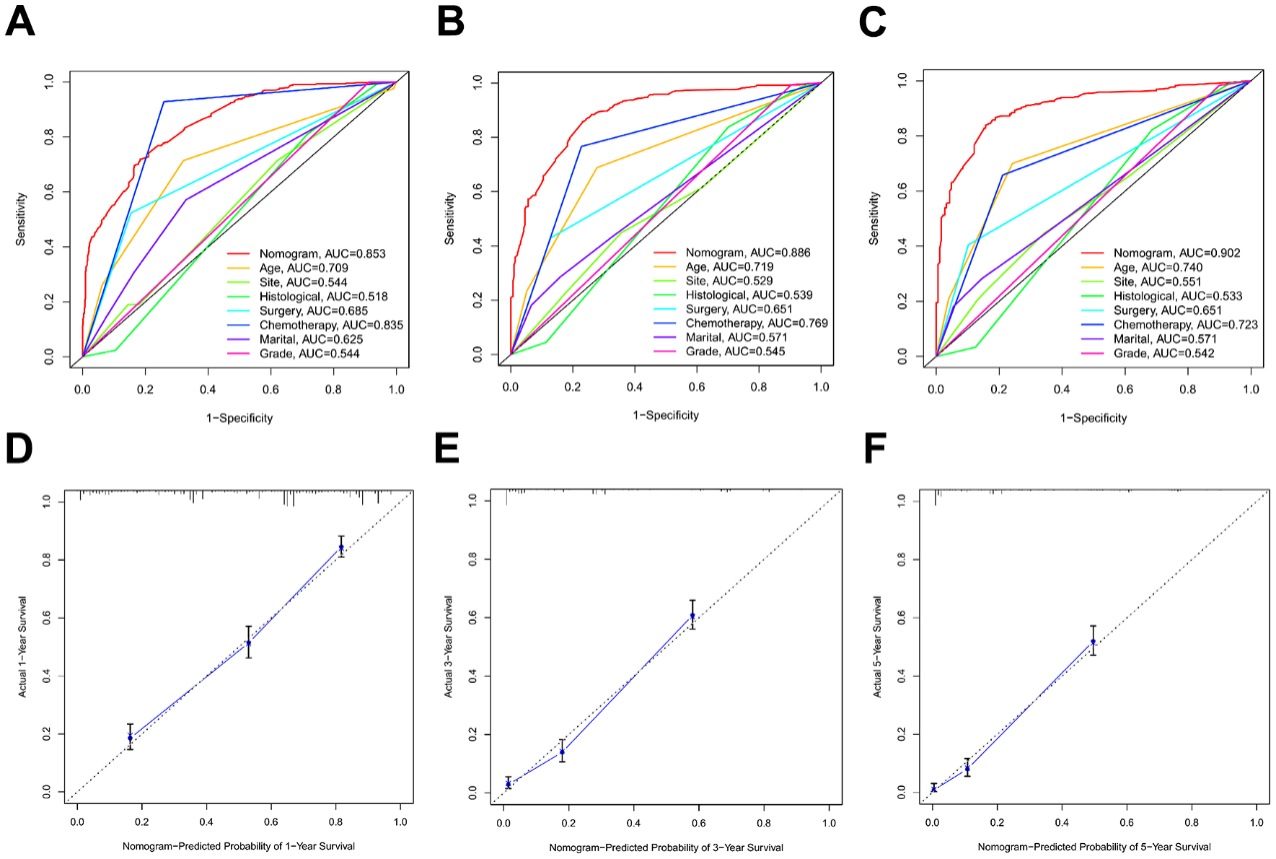
**The ROC curves and calibration plots in the validation cohort.** (**A**–**C**) ROC curves of 1-year, 3-year, and 5-year OS rates in the validation cohort. (**D**–**F**) Calibration plots of 1-year, 3-year, and 5-year OS rates in the validation cohort.

**Figure 6:**
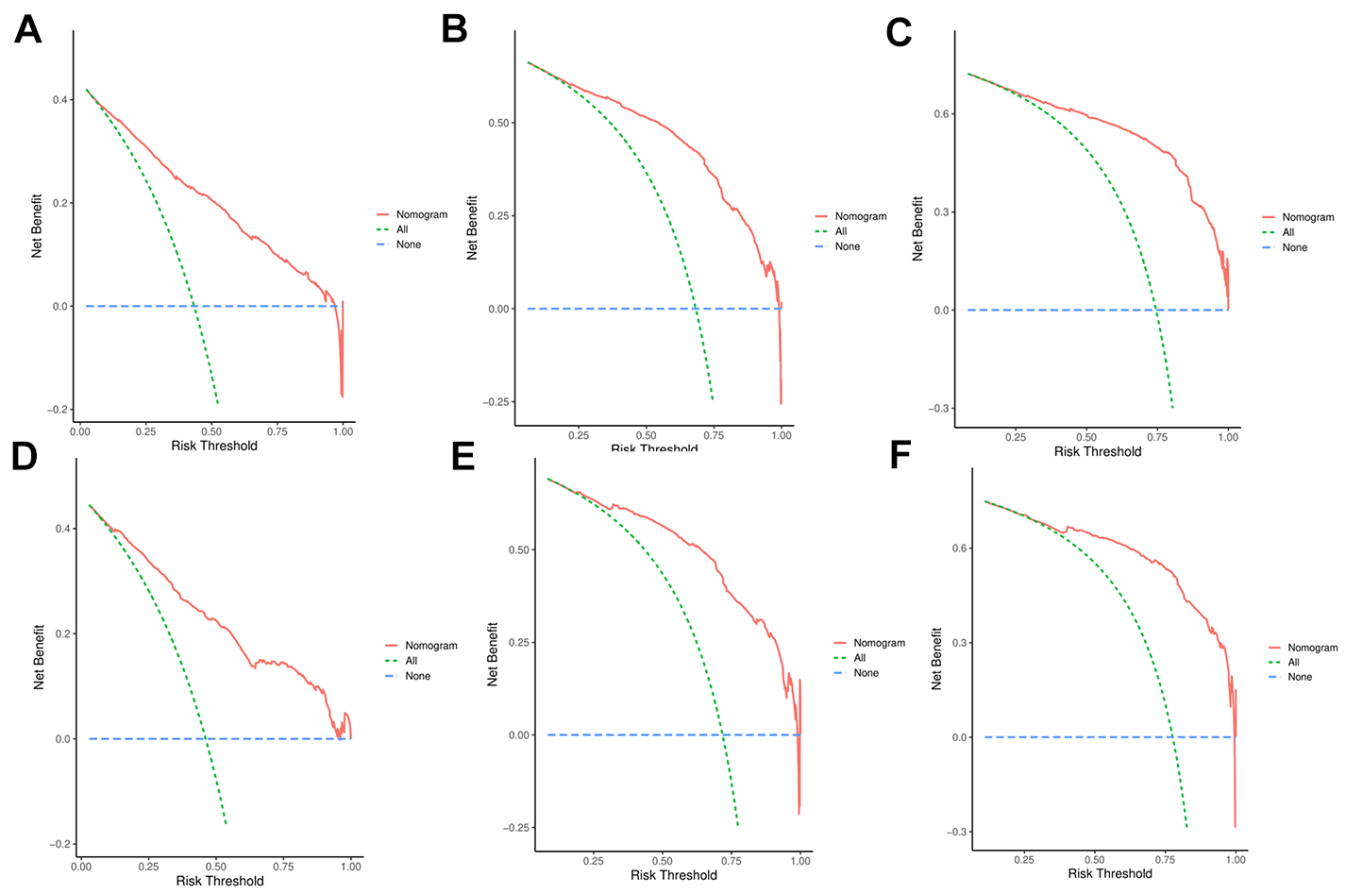
(**A**–**C**) DCA analysis predicting 1-, 3-, and 5-year overall survival (OS) in the training cohort; (**D**–**F**) DCA analysis predicting 1-, 3-, and 5-year overall survival (OS) in the validation cohort.

Discrimination and calibration techniques were used to internally validate the performance of this predictive model. Glioma patients’ C-index values in the training cohort were 0.779 (95% CI=0.769-0.789). The nomogram showed a higher prediction accuracy for OS, as demonstrated by the AUCs of each independent prognostic predictor of OS in the training set at 1-, 3-, and 5-year ROC curves, as shown in Figure 4A–4C. The training cohort predicted by the nomogram was represented by the 1-, 3-, and 5-year AUCs, which were, respectively, 0.850, 0.881, and 0.930. A strong connection was found between the observed OS and the nomogram prediction of 1-, 3-, and 5-year survival, as shown by the calibration plots of the nomogram based on age, primary site, histological type, surgery, chemotherapy, marital status, and grade (Figure 4D–4F). The C-index values in the validation cohort were 0.776 (95% CI=0.760-0.792). The validation set’s ROC curve showed AUCs for OS at1,3, and 5 years to be 0.853, 0.886, and 0.902, respectively (Figure 5A–5C). These values suggest that the nomogram exhibited good accuracy and dependability. Furthermore, the 1-, 3-, and 5-year calibration curves demonstrated remarkable agreement between the expected outcomes and the actual survival rate of patients with gliomas (Figure 5D–5F). Better clinical applications for the risk-scoring model were also highlighted by the DCA curves (Figure 6).

### Survival analyses

**Figure 7:**
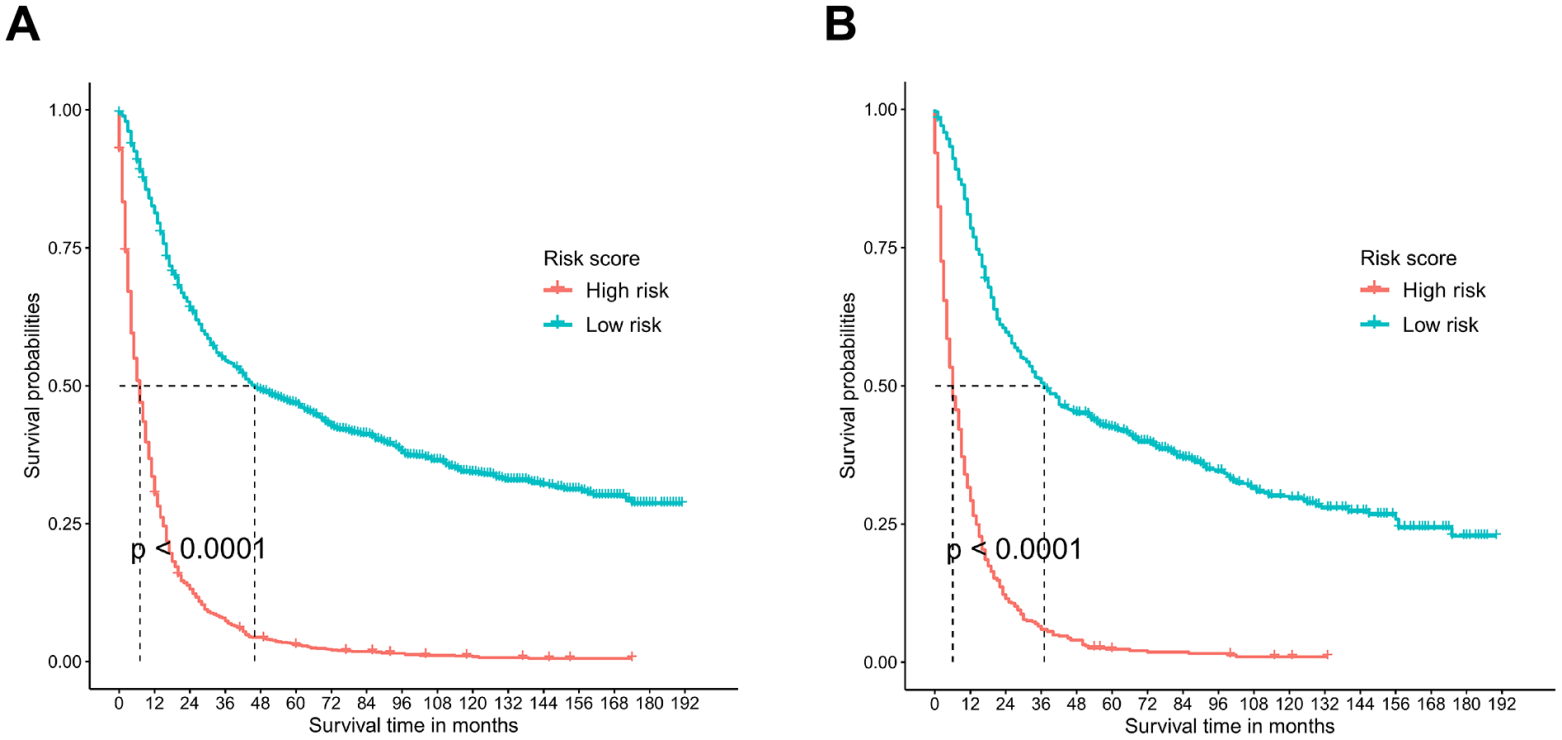
**Survival analysis performed by Kaplan-Meier analysis.** (**A**) Training cohort. (**B**) Validation cohort.

The risk scores of the patients were calculated by summing up the scores from the single items. The patients were then split into low-risk and high-risk groups based on the median. Kaplan-Meier plots were utilized for survival analysis, and the results indicated that patients in the low-risk group had a much better prognosis (P<0.001) than those in the high-risk group (Figure 7).

## DISCUSSION

Eighty-one percent of brain tumors that were malignant were gliomas, the most frequent primary intracranial tumor [14–16]. Because of its aggression, the prognosis was dismal and substantial mortality occurred. Currently, the outcome of glioma treatment is unsatisfactory, with an overall 5-year survival rate of less than 5% [17]. Gliomas have a high recurrence rate and recurrence can occur at any time after surgery, up to 36.7% at 6 months after surgery [18–20]. For this reason, it’s critical to precisely identify the variables affecting glioma patients’ prognosis in order to direct further care. Therefore, a nomogram was utilized to combine several clinical parameters in order to evaluate the patient prognosis and measure the survival of glioma patients in a more thorough manner. Glioma cases were taken from the SEER database for this investigation [21]. Seventy percent of the participants were utilized as the test set, and the remaining thirty percent were used to investigate the relationship between several characteristics that could have an impact on the patients’ overall survival. Age, primary site, histological type, surgery, chemotherapy, marital status, and grade were found to be independent prognostic factors influencing individuals with gliomas [22].

For glioma patients, age is a significant prognostic factor, particularly for those 65 years of age or older, meaning that an older patient has a higher chance of a poor prognosis. The preceding research have generally indicated that age and tumor survival are related. For instance, age was determined to be one of the most significant prognostic factors in Zhang’s study, which created and assessed a predictive nomogram model for adult ventricular glioma patients [23]. An additional finding of a Li et al. predictive model for patients with cerebellar glioma is that the age at diagnosis has a significant impact on overall survival outcomes [24]. In addition, we found that the prognosis of glioma patients varied among the different primary sites. The primary site of overlapping lesion patients had a relatively poor prognosis. This could be because glioma excision in overlapping lesions is challenging and more likely to result in poor surgical outcomes and recurrence [25]. As to histological type, glioblastoma is identified as the most prognostic type. It is well-known that glioblastoma, as a grade IV glioma, is one of the most malignant and intractable central nervous system tumors with high recurrence, low survival rate, and poor prognosis [26]. Surgical resection is the mainstay of treatment for glioma, where complete removal of the tumor as much as possible is the key to treatment [27]. The results of this study showed that surgical treatment was beneficial in improving the prognosis of patients. A study by Nsir et al. reported eight cases of ventricular glioblastoma. Compared with patients without tumor resection, three patients with total tumor resection had better prognosis [27]. Moreover, chemotherapy is also recognized as one of the prognostic factors in our study. Chemotherapy is an important tool in the treatment of glioma, especially for patients unable to undergo surgery. Chemotherapy can kill the tumor cells that remain after surgery, thus prolonging the progression-free survival and overall survival of patients [28–30]. Moreover, glioma patients’ poor prognosis was independently associated with both divorce and widowhood. This is in line with earlier research by Xie et al.l [31]. So, it is suggested that the healthcare system should be aware that patients with an aborted marriage need more social and physiological support. Compared with low-grade glioma, HRs of high-grade glioma are higher. The degree of interstitial change of the tumor tissue, which includes the local infiltration of cancer cells and the degree of differentiation, organization, and number of nuclear divisions, is used to establish the tumor grade. It can serve as a guide for prognosis and clinical treatment.

The shortcomings of our study need to be mentioned. Firstly, we only selected partial histologic types and representative sites of gliomas, so the data on rare histologic types and special sites of gliomas were not included. Secondly, detailed and comprehensive information on patients’ treatment, such as treatment modality, radiotherapy dose, and use of alternative therapies, is not available in the SEER database, which leads to an inability to fully and objectively interpret our results. In addition, patients’ disease histories are not well documented, which may affect mortality. Moreover, the nomogram calibration plot analyses of relapse-free survival could not be performed because of the lack of relevant data. Additionally, limitations in access, compatibility, and representativeness of other databases posed significant challenges. Future studies are indeed planned to incorporate additional databases for validation purposes, which we believe will strengthen the robustness and applicability of our model. Finally, because this is a retrospective study, selection bias may have occurred during the patient selection procedure. Despite the above-mentioned limitations, the distinct advantages of our study were also listed. First of all, enough sample data of cancer patients are stored in the SEER database, which may guarantee the validity of the study’s conclusions. Second, after building the prediction model, we tested it using an internal test set. The validation results point to the model’s stability and dependability.

To sum up, this study comprehensively summarized and analyzed the clinical characteristics and independent prognostic factors of glioma patients, and further constructed the nomogram prediction model of OS. The prediction model has reliable prediction efficiency, which is helpful for clinicians to assess the survival probability of glioma patients easily.

## MATERIALS AND METHODS

### Retrieve data

The clinical and survival data of glioma patients were extracted and retrieved using SEER*Stat 8.3.6 software from the SEER (http://seer.cancer.gov/) database. This database collected data from 18 registries, representing roughly 30% of the US population. Site codes and histology codes were utilized for data screening, following the guidelines provided by the third edition of the International Classification of Diseases for Oncology (ICD-O-3). Glioma patients who were diagnosed between 1975 and 2019 were retrieved based on the selection criteria. Our study’s inclusion criteria were as follows: (1) We only include the frontal lobe, temporal lobe, parietal lobe, occipital lobe, and overlapping brain lesions (C71.1, C71.2, C71.3, C71.4, and C71.8) as main primary sites of gliomas. The four main histological kinds of gliomas are oligodendroglioma (M9382, M9440, M9400, and M9450), glioblastoma, mixed glioma, and astrocytoma. Also, exclusion criteria were listed: (1) Missing data on age, race, survival time, grade, marital status, tumor size, and laterality. (2) Missing data on treatment information, including surgery, chemotherapy, and radiotherapy.

The demographics of glioma patients (age, race, gender, and marital status), disease characteristics (histological type, laterality, primary site, tumor size, and tumor grade), and treatment details (radiation, surgery, chemotherapy, and radiation sequence with surgery) were the variables that were chosen for our study. Our primary endpoint was overall survival (OS), which is measured from the time of diagnosis to death or the conclusion of the last inquiry.

### Construction and verification of nomogram

Patients were categorized into training and test groups using a 7:3 ratio. Using univariate and multivariate Cox regression analysis, independent prognostic indicators for OS in the training set were found, and the nomogram was subsequently created. The covariates in the subsequent multivariate Cox regression analysis were included if their p-value was less than 0.05 in the univariate analysis. The survival nomogram was created using the components in the multivariate analysis whose p-value was less than 0.05.

The training and validation sets were used to test the nomogram, and the ROC curves, C-index values, and calibration plots were used to assess it. To examine the degree of difference between the actual value and the anticipated value, calibration plots were employed. The discrimination was represented by the estimated C-index. Additionally, to display the sensitivity and specificity, ROC curves were plotted. Ultimately, a decision curve analysis was run to assess the clinical advantages. The R4.1.1 version was used to construct the nomogram, calibration plots, ROC curves, and DCA plots. Adobe Illustrator CS6 was used to mix and rearrange the later images.

### Statistical analysis

Age was originally classified as a continuous variable, but it was later modified to an ordered one. The χ2 test or Fisher’s exact test were used to examine the following factors: gender, ethnicity, laterality, primary site, histological type, primary, surgery, radiation, radiation sequence with surgery, chemotherapy, and marital status. These variables are classified as disordered classification variables. The Mann-Whitney U test was also used to examine ordered classification variables, such as age, tumor size, and grade. The Kaplan-Meier method was used to visually assess the differences in survival rates between the high-risk and low-risk groups. At the p <0.05 level, statistical significance was acknowledged.
